# A Single-Cell and Feeder-Free Culture System for Monkey Embryonic Stem Cells

**DOI:** 10.1371/journal.pone.0088346

**Published:** 2014-02-05

**Authors:** Takashi Ono, Yutaka Suzuki, Yosuke Kato, Risako Fujita, Toshihiro Araki, Tomoko Yamashita, Hidemasa Kato, Ryuzo Torii, Naoya Sato

**Affiliations:** 1 Discovery Molecular Pharmacology Department, Discovery Screening Center, Mitsubishi Tanabe Pharma Corporation, Yokohama, Kanagawa, Japan; 2 Division of Developmental Biology, Research Center for Genomic Medicine, Saitama Medical University, Hidaka, Saitama, Japan; 3 Research Center for Animal Life Science, Shiga University of Medical Science, Otsu, Shiga, Japan; Wellcome Trust Centre for Stem Cell Research, United Kingdom

## Abstract

Primate pluripotent stem cells (PSCs), including embryonic stem cells (ESCs) and induced pluripotent stem cells (iPSCs), hold great potential for research and application in regenerative medicine and drug discovery. To maximize primate PSC potential, a practical system is required for generating desired functional cells and reproducible differentiation techniques. Much progress regarding their culture systems has been reported to date; however, better methods would still be required for their practical use, particularly in industrial and clinical fields. Here we report a new single-cell and feeder-free culture system for primate PSCs, the key feature of which is an originally formulated serum-free medium containing FGF and activin. In this culture system, cynomolgus monkey ESCs can be passaged many times by single-cell dissociation with traditional trypsin treatment and can be propagated with a high proliferation rate as a monolayer without any feeder cells; further, typical PSC properties and genomic stability can be retained. In addition, it has been demonstrated that monkey ESCs maintained in the culture system can be used for various experiments such as *in vitro* differentiation and gene manipulation. Thus, compared with the conventional culture system, monkey ESCs grown in the aforementioned culture system can serve as a cell source with the following practical advantages: simple, stable, and easy cell maintenance; gene manipulation; cryopreservation; and desired differentiation. We propose that this culture system can serve as a reliable platform to prepare primate PSCs useful for future research and application.

## Introduction

Pluripotent stem cells (PSCs), including embryonic stem cells (ESCs) and induced pluripotent stem cells (iPSCs), have the capacity to infinitely proliferate and the ability to differentiate into a large number of cell types. Human PSCs hold great potential for applications in drug discovery, disease modeling, and regenerative medicine. Similarly, monkey PSCs have valuable applications because monkeys share many physiological similarities with humans and are well-developed primate models of neurodegenerative disorders, autoimmune diseases, reproductive biology, infectious diseases, and behavior.

On the other hand, primate PSCs are thought to be identical to rodent epiblast stem cells (EpiSCs) [1]. EpiSC-like features make it difficult to culture primate PSCs in the undifferentiated state and to regulate differentiation into desired functional cells. To realize future use, a reliable and scalable culture system for supporting primate PSC maintenance is required in addition to efficient and reproducible differentiation techniques for preparing desired cells. There are two major obstacles in conventional culture systems, which impede the industrial and clinical application of primate PSCs.

A major bottleneck is the use of feeder cells and non-defined media. Primate PSCs should be traditionally cultured on mouse embryonic fibroblast (MEF) feeder layers. Conventional culture media usually contain fetal bovine serum and/or other undefined factors. Both MEF preparation and PSC co-culture with MEF feeder cells are laborious and time-consuming. MEF feeder systems limit the reproducibility and large-scale preparation of primate PSCs. In addition, MEF feeders and culture media contain several unknown contaminants, which give rise to unstable experimental conditions and results varying from batch-to-batch and laboratory-to-laboratory. To date, several feeder-free culture systems for primate PSCs have been reported [2]–[10]. Most culture systems are based on an MEF-conditioned medium (MEF-CM) or commercial media such as mTeSR1 [11], [12] and StemPro [13] and/or animal-derived products such as Matrigel, a complex mixture of matrix proteins [14]. Commercial media utilize several growth factors or chemicals that can mimic growth factor signaling to promote the growth of primate PSCs. Therefore, defined media that can be adapted to specific needs are essential for functional studies of the self-renewal potential and differentiation-inducing property in PSCs. However, major compositions of these commercial media are either unknown or rather complex. Furthermore, the widespread use of Matrigel as a culture substrate is potentially problematic [15]. Matrigel is not an optimal substrate because it is derived from Engelbreth–Holm–Swarm mouse tumors and contains many unknown components [14]. Thus, the development of a feeder-free culture system involving a defined medium is recommended to potentiate the practical use of primate PSCs.

Primate PSCs are generally cultured as colonies and are harvested as small cell clumps by partial dissociation using either enzymatic or mechanical methods. It is difficult to precisely control the appropriate dissociation of primate PSCs during each passage, and variation in the quality and size of colonies depends on the handling skills of experimenters in laboratories. The quality of colonies plays a critical role in the downstream applications. Therefore, it is arduous to efficiently direct the desired differentiation of primate PSCs in a reproducible and scalable manner. Cryopreservation of primate PSC clumps requires specialized equipment and apparatus, which has severely limited their utility. Furthermore, the requirement to handle primate PSCs as cell clumps hampers their efficient use for genetic manipulation research in gene transfer and clonal analysis. Taken together, it seems that primate PSCs allowing a stable single-cell passage would serve as a useful cell source for genetic manipulation and cryopreservation experiments as well as for large-scale PSC preparation.

Although many approaches to overcome these obstacles have been reported, these are insufficient for the practical use of primate PSCs.

In this study, we report a novel single-cell passage and feeder-free culture system which allows monkey ESCs to be maintained simply and stably without any complicated cell manipulation. Further, the culture system can be made available for human ESCs and iPSCs.

## Materials and Methods

### Ethics statement

All experimental procedures involving cynomolgus monkeys were approved by the Animal Care and Use Committee of Shiga University of Medical Science (Permit Number: 2011-10-5H). Mature cynomolgus monkeys were housed individually in cages that were 500 mm wide by 800 mm deep by 800 mm high. Light cycle was 12 h of artificial light from 8:00 to 20:00. Temperature and humidity in the animal room were maintained at 25±2°C and 50±5%, respectively. Each animal was fed 20 g/kg/day of commercial pellet monkey chow (CMK-1, CLEA, Japan) in the morning, supplemented with 20–50 g of sweet potato and half a banana in the afternoon. Water was supplied ad libitum by an automatic supplier. No monkeys were sacrificed in this study. The animal welfare and steps taken to ameliorate suffering were in accordance with the recommendations of the Weatherall report, “The use of non-human primates in research”.

All experimental procedures involving mice were approved by the Committee on the Ethics of Animal Experiments of UNITECH Co., Ltd. (Permit Number: AGRTNB-120412-30) and carried out in strict accordance with the recommendations in the Guide for the Care and Use of Laboratory Animals of the National Institutes of Health. All surgery was performed under sodium pentobarbital anesthesia, and all efforts were made to minimize suffering.

### MT-fCFA medium

The MT-CDM medium is a chemically defined medium based on the composition of the CDM medium [4], [16]. The original composition of the MT-CDM medium was DMEM/F-12, GlutaMAX (Gibco) supplemented with 7 µg/ml Insulin (Sigma), 15 µg/ml transferrin (Sigma), 450 µM monothioglycerol (Sigma), 1× lipid concentrate (Gibco), and 5 mg/ml bovine serum albumin (Sigma). The MT-fCFA medium is a fortified MT-CDM medium supplemented with 15 ng/ml FGF2 (Peprotech, 100-18B), 10 ng/ml activin A (R&D Systems, 338-AC), 14 ng/ml sodium selenium (Sigma, S5261), 64 µg/ml L-ascorbic acid-2-phosphate magnesium (Wako, 323–44822), 1× non-essential amino acids (Gibco, 11140–050), and 10 ng/ml heparin sodium salt (Sigma, H4784).

### Single-cell and feeder-free culture system

Monkey ESCs were cultured at 37°C and 5% CO_2_ in the MT-fCFA medium. For passaging, the cells were washed twice with phosphate-buffered saline (PBS) and were treated with 0.01% trypsin/0.004% EDTA (Sigma) for 2 min, followed by pipetting to ensure single-cell dissociation. The single dissociated cells were then mixed with 250 µg/ml of trypsin inhibitor (Gibco) to inactivate trypsin, centrifuged at 180×g for 5 min, and resuspended in the MT-fCFA medium containing 2.4 µM thiazovivin (Wako) and 4.7 µg/ml human fibronectin (BD Biosciences). The cells were plated on collagen type I-coated dishes (IWAKI) at a dilution ratio of 1∶8–1∶10. Cells routinely received fresh medium every day and were passaged when 80–90% confluence was reached, which normally occurred every 2–3 days.

For cryopreservation, single dissociated cells were suspended in STEM-CELLBANKER (ZENOAQ, BLC-3S) [17] and directly frozen at −80°C without a programmed freezer in accordance with the manufacturer's instructions.

### Growth curve assay

After plating, ESCs were imaged using a real-time video imaging system (IncuCyte ZOOM, Essen BioScience). Growth curves were built from confluence measurements using the IncuCyte ZOOM software. Frames were captured at 30-min intervals from 50 separate regions per 100-mm dish or from 16 separate regions per well in a 6-well plate using a 10× objective. Values for each time point were averaged across all separate regions.

### *In vitro* differentiation

To induce differentiation into the neuroectoderm, ESCs were cultured for 4 days in the MT-CDM medium in the presence of 10 µM SB431542 (SB; Wako), 1 µM PD0325901 (PD; Wako), and 1 µM dorsomorphin (DM; Wako). The resulting cells were transferred to non-adherent conditions for 7 days in the MT-fCF medium (MT-fCFA medium without activin) in the presence of 10 µM SB and were then plated back on a poly-D-lysine/laminin-coated plate for 32–39 days in Neurobasal medium (Invitrogen) supplemented with B-27 Supplement Minus Vitamin A (Invitrogen) and 1 µM cyclopamine (Wako) to promote the generation of cortical neurons. To induce mesendodermal differentiation, ESCs were cultured in the MT-CDM medium in the presence of 10 ng/ml activin and 1 µM DM for 4 days

### Immunocytochemistry and alkaline phosphatase staining

Cells were fixed with 4% (w/v) paraformaldehyde in PBS for 10 min, permeabilized with 0.1% (v/v) Triton X-100 in PBS for 15 min, and blocked in 1% normal donkey serum for 1 h. Cells were incubated with primary antibodies for 1 h, washed with PBS, and incubated with appropriate Alexa Fluor-conjugated secondary antibodies for 1 h. Nuclei were counterstained with 20 µM Hoechst 33342 (Dojindo, H342) for 10 min. Primary antibodies were as follows: Nanog (1∶800; Cell Signaling, 4903), Oct4 (1∶100; SantaCruz, sc-5279), Sox2 (1∶100; R&D Systems, MAB2018), SSEA-4 (1∶100; Stemgent, 09-0006), Tra-1-60 (1∶100; Stemgent, 09-0010), Tra-1-81 (1∶100; Stemgent, 09-0011), Pax6 (1∶200; BD Biosciences, 561462), Sox1 (1∶100; Stemgent, 09-0084), T/Brachyury (1∶200; SantaCruz, sc-17745), Tuj1 (1∶500; Millipore, MAB1637), MAP2 (1∶500; Millipore, AB5622), DCX (1∶500; Abcam, ab18723), and CTIP2 (1∶500; Abcam, ab18465). Alkaline phosphatase staining was performed using the HNPP Fluorescent Detection Set (Roche).

### Flow cytometric analysis

Cells were fixed and permeabilized with Fixation/Permeabilization buffer (eBioscience) overnight at 4°C. After washing three times in FCM buffer (PBS containing 1% fetal calf serum and 0.1% azide), non-specific protein binding in permeabilized cells was blocked by incubation in FCM buffer containing 2% normal rat serum for 15 min at room temperature (RT). Cells were co-stained with Alexa Fluor 647-conjugated anti-Nanog (1∶15; Cell Signaling, 5448), Alexa Fluor 488-conjugated anti-Oct4 (1∶5; BD Biosciences, 560253), and phycoerythrin (PE)-conjugated anti-Sox2 (1∶10; R&D Systems, IC2018P) or the corresponding isotype controls in FCM buffer. Flow cytometric analysis was performed on BD LSRFortessa equipped 405-nm, 488-nm, and 633-nm lasers. The data acquired were analyzed using the FlowJo software (Tree Star).

### Karyotype analysis

Cells were treated with 60 ng/ml KaryoMAX Colcemid Solution (Invitrogen) for 6 h to arrest the cells in metaphase. Next, the cells were harvested by trypsin treatment. After two washes in PBS, cells were gently resuspended in a hypotonic (75 mM) KCl solution and incubated at 37°C for 20 min. Cells were then centrifuged and resuspended in cold methanol:acetic acid 3∶1 (v/v) for fixation. After fixation, cells were centrifuged again and resuspended in a small volume (200 µl/10^6^ cells) of the same cold methanol:acetic acid solution. These cells were spread onto glass slides, allowed to dry, and stained with KaryoMAX Giemsa Stain (Invitrogen). After washing the slides with water, a minimum of 10 chromosome spreads were analyzed.

### Teratoma formation analysis

Teratoma formation analysis was conducted at the specific pathogen-free animal facility of UNITECH Co., Ltd. (Chiba, Japan). In brief, the single dissociated ESCs (1–2×10^6^ cells/site) were harvested by trypsin treatment and suspended in 100 µl of the MT-fCFA medium supplemented with 2.4 µM thiazovivin and 100 µl of BD Matrigel, and were injected into the testis and subcutaneous spaces of 7-week-old severe combined immunodeficient (SCID) male mice. After 8–10 weeks, the mice were sacrificed and the tumors formed were harvested, fixed in 10% neutral buffered formaldehyde, embedded in paraffin, sectioned, and analyzed for various differentiated cell types and structures by hematoxylin and eosin staining.

### Gene transfer and clonal isolation

The plasmid pCAG-GFP2, which carries CAG promoter-driven GFP2 green fluorescent protein cDNA and a neomycin resistance gene, was used for gene transfer experiments.

CMK6_SFF_ cells (8×10^5^ cells) were transfected with 2 µg of pCAG-GFP2 using Human Stem Cell Nucleofector Solution 1 (Amaxa) and the A-013 program, as per the manufacturer's instructions. A working dose of the neomycin analog G418 for selection in CMK6_SFF_ cells was established using a kill curve. The lowest dose (80 µg/ml) that would completely kill non-transfected CMK6_SFF_ cells over 5 days was used. After 7–10 days of gene transfer, G418-resistant clones derived from single cells were selected and allowed to propagate in the MT-fCFA medium containing 80 µg/ml G418.

### Calcium influx assay

Cells were loaded with the assay dye solution (PBX Calcium Assay Kit, BD Biosciences) for 1 h. N-methyl-D-aspartate (NMDA; SIGMA) and glycine (WAKO) were added using the FDSS6000 System (Hamamatsu) following a 10-s baseline determination. NMDA receptor antagonist, MK-801 (SIGMA) or ifenprodil (WAKO), was incubated for 10 min before the addition of NMDA and glycine. Fluorescence data were collected at 1-s intervals for 80 s and were analyzed using the FDSS6000 software. All experiments were performed at RT.

## Results

### Adaptation to the single-cell and feeder-free culture system of cynomolgus monkey ESCs

To develop a novel single-cell and feeder-free culture system for primate PSCs, we formulated the original MT-fCFA medium, a chemically defined medium supplemented with FGF and activin (see *Materials and Methods*). We first attempted the adaptation of monkey ESCs previously established on conventional culture to the MT-fCFA medium.

Cynomolgus monkey ESCs, CMK6 cell line [18], were completely dissociated to single cells by mild trypsin treatment and then seeded on collagen type I-coated dishes using the MT-fCFA medium containing a ROCK inhibitor thiazovivin for preventing cell death [19], [20] and fibronectin for promoting cell attachment to the culture dish bottom [4].

**Figure 1:**
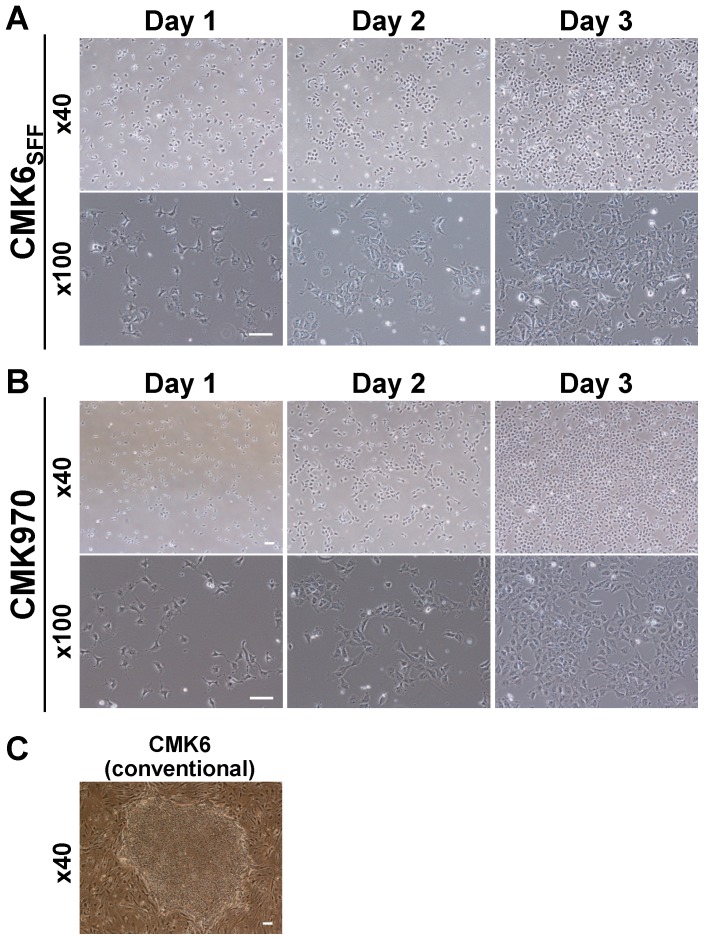
Morphology of monkey ESCs grown under the MT-fCFA culture condition. A, B. CMK6_SFF_ (A) and CMK970 (B) cells maintained in the single-cell and feeder-free culture system with the MT-fCFA medium grow as a uniform monolayer with a high proliferation rate. Each cell shows an undifferentiated morphology with a high nucleus-to-cytoplasm ratio. C. CMK6 cells maintained under the conventional culture condition with MEF feeders. *Scale bar* = 100 µm.

During the first passage, most of the dissociated cells adhered to the culture dish surface and started proliferating, although a small population of dead cells was observed. After several passages, the cells stably propagated as a uniform monolayer without colony formation (Figure 1A). We termed these cells as CMK6_SFF_, which indicates that the CMK6 cells adapted to the single-cell and feeder-free (SFF) culture system, in order to distinguish them from the cells grown under conventional culture conditions (Figure 1C). CMK6_SFF_ cells grown under the MT-fCFA culture condition exhibited a higher proliferation rate than those grown under the conventional culture conditions (Table 1) and had undifferentiated cell morphology with a high nucleus-to-cytoplasm ratio (Figure 1A).

**Table 1:** Passage frequency and split ratio of monkey ESCs grown under the MT-fCFA culture condition.

Cell	Culture condition	Passaging Frequency (Days)	Split Ratio
CMK6	20% KSR, DMEM/F12, Colony on MEF	3–4	1∶4–5
CMK6_SFF_	MT-fCFA, Single-cell, Feeder-free	2–3	1∶4–8
CMK970	MT-fCFA, Single-cell, Feeder-free	2–3	1∶5–10

CMK6_SFF_ indicates the CMK6 cells adapted to single-cell and feeder-free condition. CMK970 is a cell line established under the SFF condition immediately after ICM isolation.

### Establishment of monkey ESC line under the MT-fCFA culture condition

We attempted to establish a new monkey ESC line under the MT-fCFA culture condition. This trial was conducted with the inner cell mass of cynomolgus monkey blastocysts obtained by intracytoplasmic sperm injection, as described previously [21], using the MT-fCFA medium instead of the conventional medium. The newly generated cell line, termed as CMK970 cells, propagated as a uniform monolayer without colony formation, similar to CMK6_SFF_ cells (Figure 1B). CMK970 cells proliferated at a higher rate than the conventional colony-forming CMK6 cells (Table 1).

Taken together, these data indicate that the culture system universally enables monkey ESCs to be adapted and maintained in single-cell and feeder-free culture.

### Characterization of monkey ESCs under the MT-fCFA culture condition

To evaluate the long-term stability of this MT-fCFA culture condition, monkey ESCs were passaged multiple times as described above. During the prolonged maintenance, CMK6_SFF_ and CMK970 cells expanded to 4–8- and 5–10-fold, respectively, every 2–3 days (Table 1). Over multiple passages, there were no significant changes in cell morphology and growth rate. We confirmed that both CMK6_SFF_ and CMK970 cells could be cultured for more than 80 passages (data not shown).

**Figure 2:**
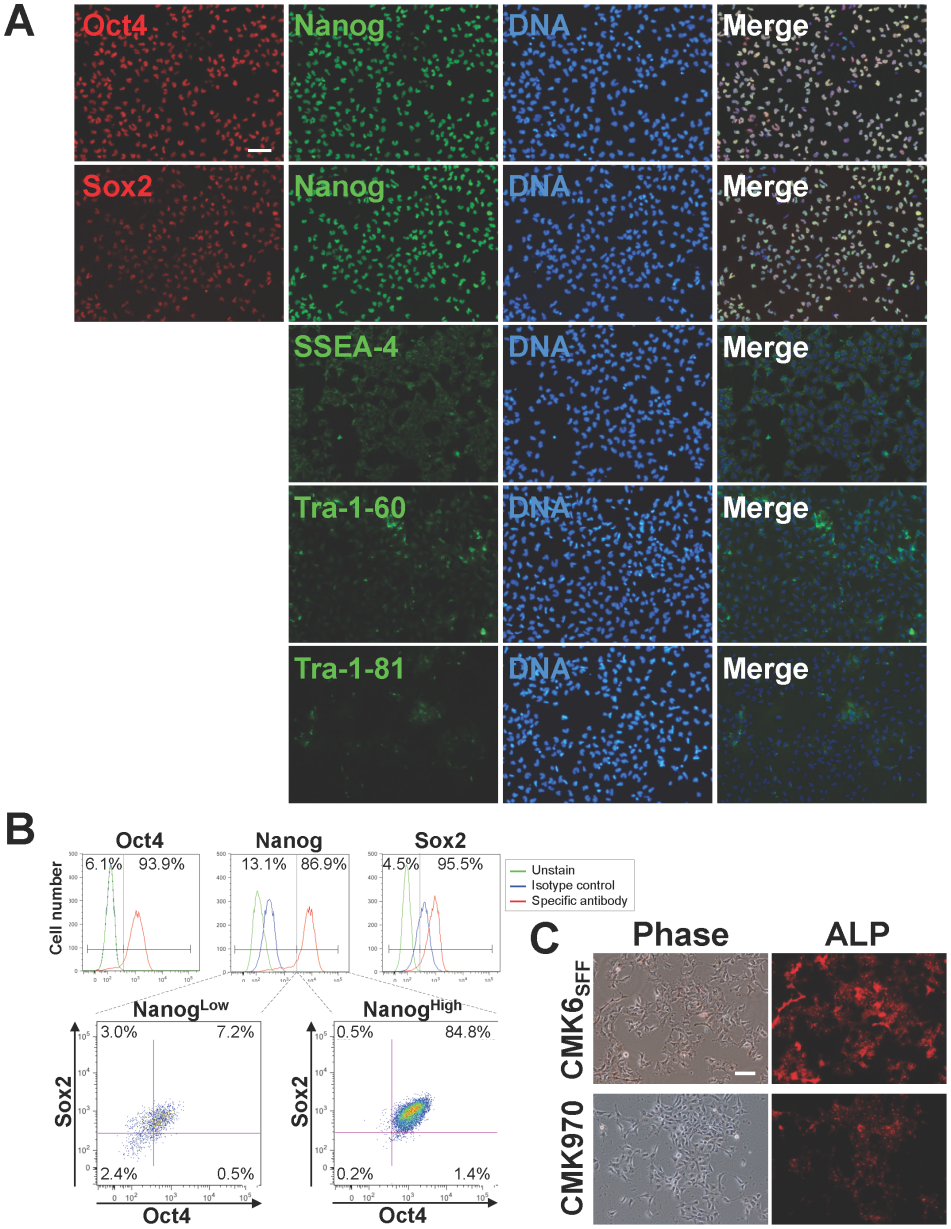
Pluripotency of Monkey ESCs grown under the MT-fCFA culture condition. A. Immunocytochemical analyses show that CMK6_SFF_ cells (P33) have a characteristic expression pattern of typical pluripotency factors, Nanog, Oct4, and Sox2 as well as that of cell surface markers, SSEA-4, TRA-1-60, and TRA-1-81, indicating their undifferentiated and pluripotent state. *Scale bar* = 100 µm. B. Flow cytometric analysis of Nanog, Oct4, and Sox2 co-expressing CMK6_SFF_ cells (P35) under the MT-fCFA culture condition. Cells were co-stained with Alexa Fluor 647-conjugated anti-Nanog, Alexa Fluor 488-conjugated anti-Oct4, and PE-conjugated anti-Sox2 or the corresponding isotype controls. C. CMK6_SFF_ (P37) and CMK970 (P31) cells are positive for alkaline phosphatase activity. ALP, alkaline phosphatase. *Scale bar* = 100 µm.

To verify the undifferentiated state of monkey ESCs under the MT-fCFA culture condition, we examined the expression of undifferentiated ESC markers. Immunocytochemical analyses showed that both CMK6_SFF_ and CMK970 cells had a characteristic expression pattern of typical pluripotency factors such as Nanog, Oct4, and Sox2 as well as that of cell surface markers, including SSEA-4, TRA-1-60, and TRA-1-81 (Figure 2A and S1A). Flow cytometric analyses also revealed that 84.8% CMK6_SFF_ cells and 90.2% CMK970 cells are Nanog/Oct4/Sox2 triple-positive (Figure 2B and S1B). Both CMK6_SFF_ and CMK970 cells retained the alkaline phosphatase activity known as one of the characteristics of potential PSCs (Figure 2C).

**Figure 3:**
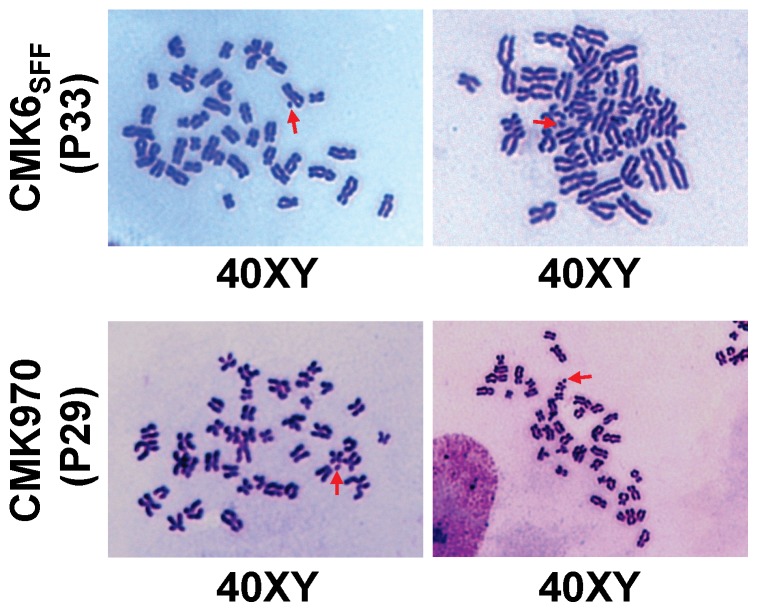
Karyotype analysis. CMK6_SFF_ and CMK970 cells retain the normal karyotype, male 40 XY. Red arrows indicate the Y chromosome.

It has been reported that single-cell passage and/or feeder-free culture conditions of primate PSCs often result in karyotypic abnormality during prolonged maintenance [22], [23]. Karyotype analysis showed that both CMK6_SFF_ cells at 33rd passage and CMK970 cells at 29th passage had the normal karyotype, male 40 XY (Figure 3), suggesting the genomic stability under the prolonged MT-fCFA culture condition.

### *In vivo* pluripotency

**Figure 4:**
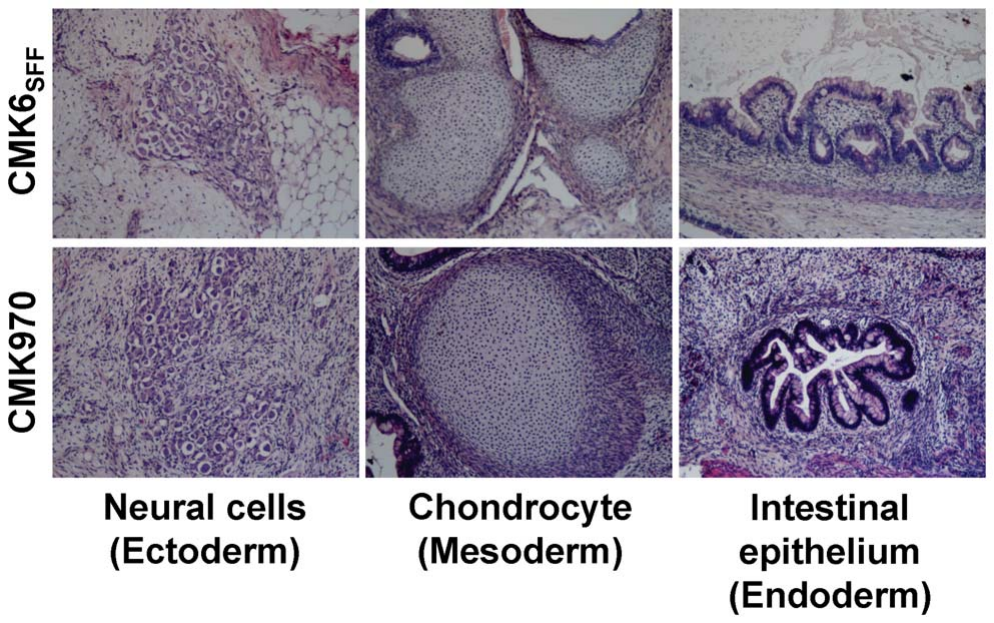
Teratoma formation. Teratomas consisting of the three germ layers developed from CMK6_SFF_ and CMK970 cells under the MT-fCFA culture condition. Cells were injected into the testis and subcutaneous space of SCID mice. After 8–11 weeks, the fate of the cells was analyzed. Hematoxylin and eosin staining showed differentiation into various tissues, including neural cells (ectoderm), chondrocyte (mesoderm), and intestinal epithelium (endoderm).

To examine the *in vivo* pluripotency of monkey ESCs under the MT-fCFA culture condition, a teratoma formation assay was performed. Approximately 1–2×10^6^ cells of single dissociated CMK6_SFF_ and CMK970 cells were individually transplanted into SCID mice. After 8–11 weeks, we observed tumor formation in both ESC lines. Histological examination revealed that the cells formed teratomas consisting of cell types of all three germ layers: neural cells (ectoderm), chondrocyte (mesoderm), and intestinal epithelium (endoderm) (Figure 4). These data suggest that both CMK6_SFF_ and CMK970 cells possessed the developmental potential to differentiate into any cell types of the three germ layers, suggesting their competence of *in vivo* pluripotent differentiation.

Taken together, these data indicate that both ESC lines, CMK6_SFF_ and CMK970, maintained their undifferentiated and pluripotent state during multiple passages under the MT-fCFA culture condition.

### Gene manipulation

Next, to determine whether this culture system allows for simple and efficient gene manipulation of PSCs, we performed clonal isolation of gene transfected cells using conventional electroporation (Amaxa™ Nucleofector technology).

**Figure 5:**
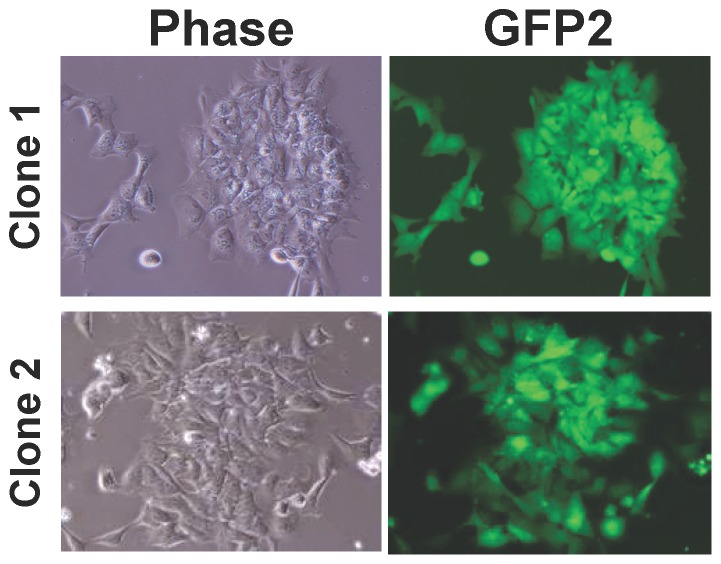
Clonal isolation after gene transfer in CMK6_SFF_ cells. The plasmid pCAG-GFP2, which carries the CAG promoter-driven GFP2 green fluorescent protein cDNA and a neomycin resistance gene, was used for the gene transfer experiment. CMK6_SFF_ cells were transfected with pCAG-GFP2 using Human Stem Cell Nucleofector Solution 1 (Amaxa) and the A-013 program following the manufacturer's instructions. After 7–10 days of the gene transfer, G418-resistant colonies derived from single cells were selected and propagated in the MT-fCFA medium containing 80 µg/ml G418.

CMK6_SFF_ cells were electroporated with pCAG-GFP2 carrying the constitutive GFP2-expression gene without optimizing transfection conditions, according to the manufacturer's instructions. After G418 selection for the transfectants, many individual transfected clones derived from single cells were readily obtained, each of which stably expressed GFP2 fluorescence (Figure 5). This result indicates that the simple and efficient gene transfer experiment was successful for monkey ESCs, which are capable of single-cell passage.

### Cryopreservation

**Figure 6:**
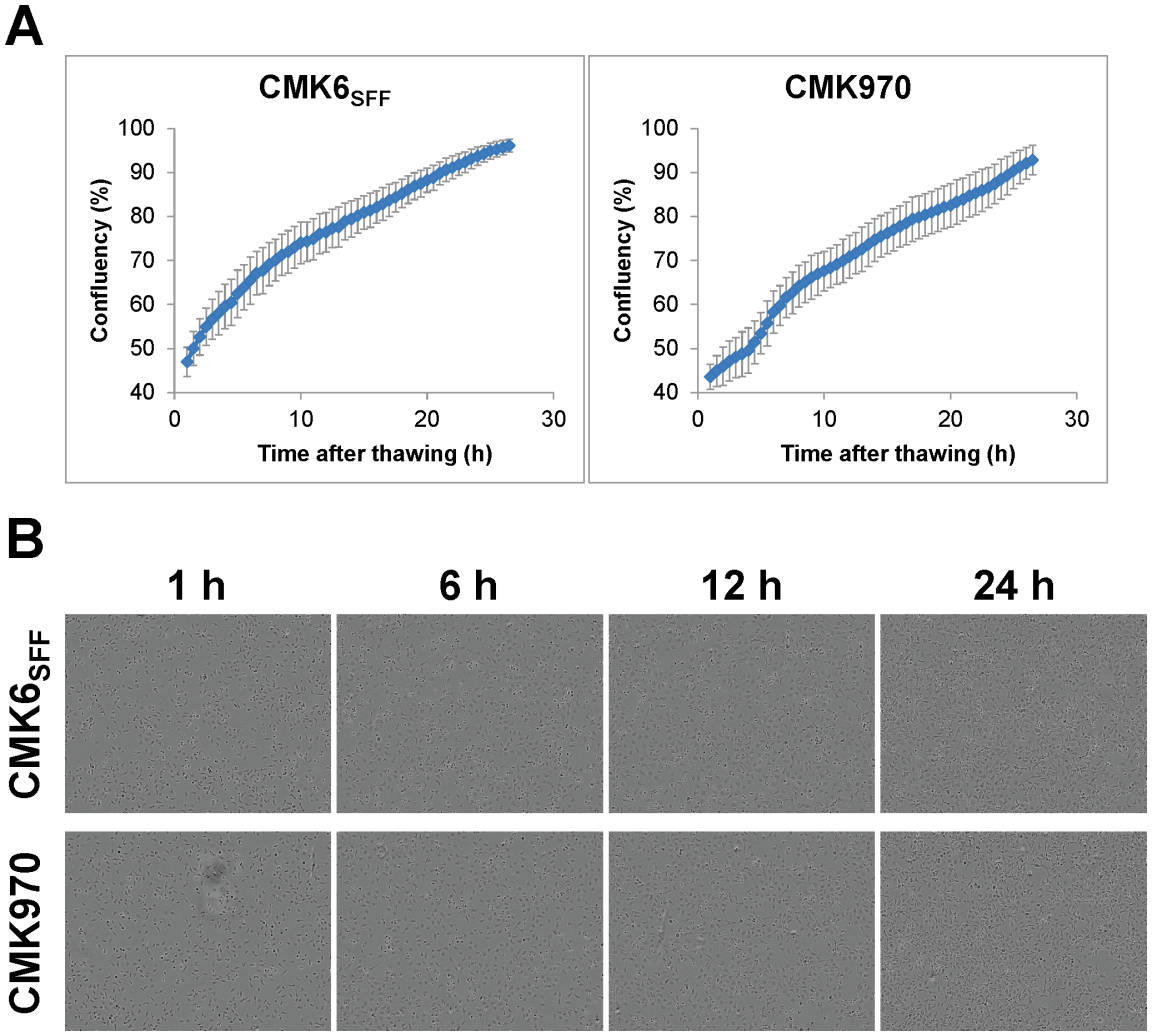
Growth curves of CMK6_SFF_ and CMK970 cells after thawing. A. Growth curves of CMK6_SFF_ and CMK970 cells after thawing were constructed using the IncuCyte ZOOM System, where the growth curves were built from confluence measurements acquired at 30-min intervals. Values from each time point were averaged across 50 separate regions. Error bars represent 1 SD about the mean for 50 independent regions. B. Representative phase images of CMK6_SFF_ and CMK970 cells at different time points after thawing.

Cryopreservation for primate PSCs is generally too time-consuming and labor-intensive because of poor survival after single-cell dissociation. In our study, single dissociated CMK6_SFF_ and CMK970 cells were directly frozen using the commercially available freezing medium STEM-CELLBANKER [17]. The protocol for cryopreserving cells is very simple and requires no specialized equipment such as a programmed freezer. To check the viability of the cells after thawing, growth kinetics were analyzed using a real-time video imaging system. As the result, after thawing, the cells quickly adhered to the dishes, showed good viability, and actively proliferated with the same corresponding morphological characteristics observed before cryopreservation (Figure 6).

These results indicate that the commercially available simple cryopreservation method is applicable to long-term storage of large quantities of primate PSCs.

### *In vitro* differentiation potential

To address the controllable and reproducible induction of differentiation, we evaluated the *in vitro* multi-directional differentiation potential of CMK6_SFF_ cells using small molecule inhibitors of growth factor signaling.

**Figure 7:**
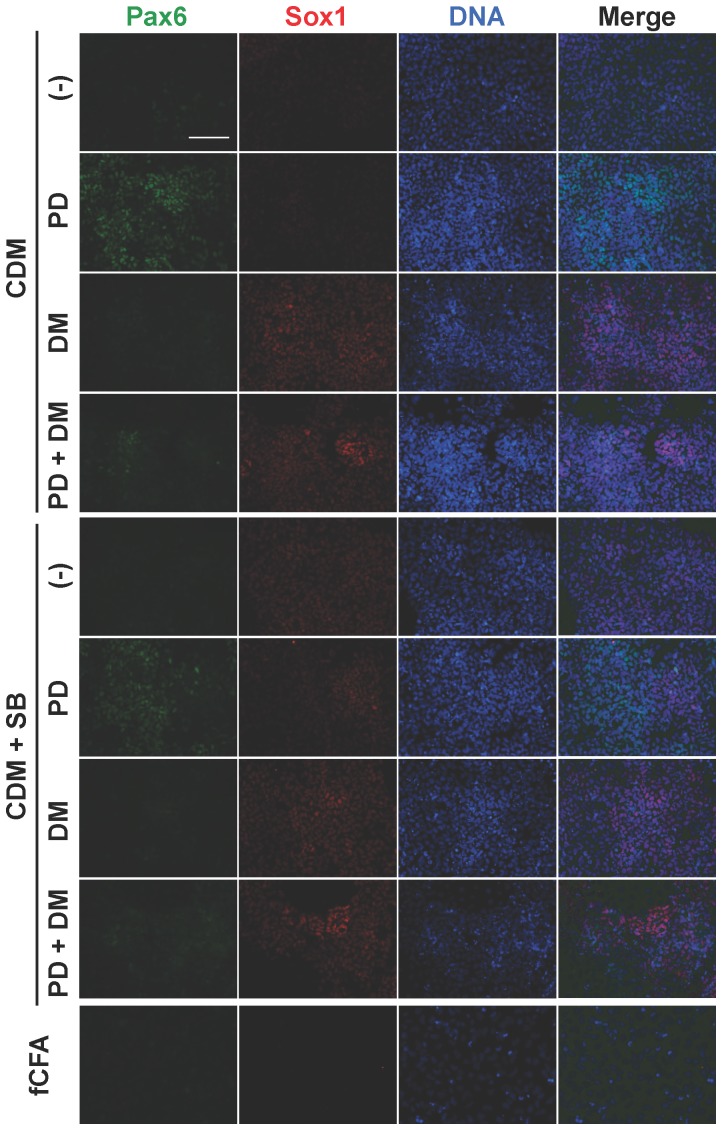
Directional differentiation to neuroectodermal cell lineages. CMK6_SFF_ cells were treated in the MT-CDM medium in the presence or absence of 10 µM SB, 1 µM PD, and 1 µM DM for 4 days. A combination of SB and PD and/or DM in the MT-CDM medium generated the cells that highly expressed the neuroectoderm markers Sox1 and/or Pax6. SB, SB431542 (TGF-β inhibitor). PD, PD0325901 (MEK inhibitor). DM, dorsomorphin (BMP inhibitor). *Scale bar* = 100 µm.

The effect of inhibiting activin, FGF, and BMP signaling pathways was analyzed in the MT-CDM medium (MT-fCFA medium without FGF, activin, and other additives) using the activin signaling inhibitor SB431542 (SB) [24], [25], FGF signaling inhibitor PD0325901 (PD) [26], and BMP signaling inhibitor dorsomorphin (DM) [27]. The expression of pluripotency markers Nanog and Oct4, the pluripotency and neuroectoderm progenitor marker Sox2, the mesendoderm marker T/Brachyury, and the neuroectoderm markers Pax6 and Sox1 was analyzed by immunocytochemistry after a 4-day treatment. The expression of Nanog and Oct4 markedly decreased by activin removal or SB addition, while that of Sox2 was maintained (Table 2, rows 1–8), confirming that activin signaling is necessary for the maintenance of the pluripotent status and its inhibition induces differentiation toward neuroectoderm. A combination of SB and PD and/or DM treatment induced high expression of Sox1 and/or Pax6 (Figure 7 and Table 2, rows 6 and 8). Taken together, these results demonstrate that complete inhibition of FGF and/or BMP signaling with inhibition of activin signaling is sufficient for generating a population of neuroectoderm progenitors.

**Table 2:** Directional differentiation to neuroectodermal or mesendodermal cell lineages.

	Conditions			Pluri.	Mesendo.		Neuroecto.		
	Activin	FGF	BMP	Nanog	Oct4	T	Sox2	Pax6	Sox1
1	-	-	-	-	-	-	++	±	-
2	-	PD 1	-	-	-	-	+	++	±
3	-	-	DM 1	-	-	±	++	±	++
4	-	PD 1	DM 1	-	-	±	++	+	+
5	SB 10	-	-	-	-	-	++	±	+
6	SB 10	PD 1	-	-	-	-	+	++	+
7	SB 10	-	DM 1	-	-	-	++	±	++
8	SB 10	PD 1	DM 1	-	-	-	++	+	++
9	Act 10	-	-	+	++	+	++	-	-
10	Act 10	PD 1	-	++	++	-	++	-	-
11	Act 10	-	DM 1	+	++	++	+	-	-
12	Act 10	PD 1	DM 1	+	+	-	+	-	-
13	Act 10	FGF 15	-	++	++	+	++	-	-
14	Act 10	FGF 15	DM 1	++	++	+	++	-	-
15	Act 10	FGF 15	BMP 10	+	++	+	+	-	-

CMK6_SFF_ cells were treated in the MT-CDM medium in the presence or absence of 10 µM SB, 1 µM PD, 1 µM DM, and 10 ng/ml activin for 4 days. SB, SB431542 (TGFβ inhibitor). PD, PD0325901 (MEK inhibitor). DM, dorsomorphin (BMP inhibitor). Act, activin. Pluri, pluripotency marker. Mesendo, mesendoderm marker. Neuroecto, neuroectoderm marker.

Meanwhile, addition of activin alone was sufficient to maintain the expression of Oct4 (Table 2, row 9). DM treatment in the presence of activin induced the expression of T/Brachyury, whereas the expression of Nanog and Sox2 did not disappear completely (Figure S2 and Table 2, row 11). In addition, cells expressing T/Brachyury were not observed with PD treatment in the presence of activin (Table 2, rows 10 and 12), confirming the importance of FGF signaling in the commitment of PSCs toward the mesendoderm. These data show that the modulation of activin, FGF, and BMP signaling can lead to the differentiation of the mesendoderm.

Taken together, these findings suggest that the culture system allows for the controllable and reproducible induction of differentiation by regulating the combination of activin, FGF, and BMP signaling using small molecule inhibitors.

### Neuronal differentiation *in vitro*

**Figure 8:**
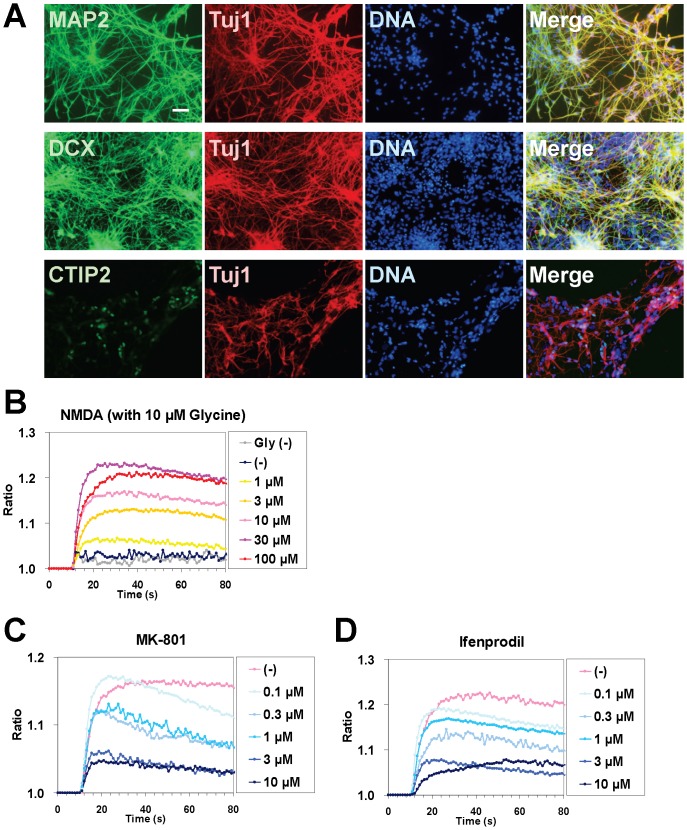
Neuronal differentiation. CMK6_SFF_ cells were differentiated into cortical neurons. A. Immunocytochemical analysis. Scale bar = 50 µm. B–D. NMDA-induced Ca^2+^ influx. NMDA (with 10 µM glycine) induced a concentration-dependent increase in [Ca^2+^]_i_ (B). MK-801 (C, NMDA receptor antagonist) and ifenprodil (D, NR2B-specific antagonist) decreased NMDA (10 µM)-induced Ca^2+^ influx.

Finally, we evaluated whether ESC-derived neuroectodermal cells retain the capacity to differentiate into cortical neurons. The cells were cultured as floating neurospheres for 7 days. Six weeks after plating, we examined the expression of neuronal markers by immunocytochemistry. A high percentage of cells expressed the neuronal markers Tuj1, MAP2, and doublecortin, as well as the subcerebral projection neuron marker CTIP2 (Figure 8A).

In cortical neurons, NMDA receptors play an important role in facilitating learning and memory [28]. We focused on NR2B subunit-containing NMDA receptors, whose hypofunction is involved in the pathogenesis of schizophrenia. The functional role of the receptors was assessed by intracellular calcium influx experiments. The studies revealed that NMDA with 10 µM glycine induced a concentration-dependent increase in the intracellular calcium concentration (Figure 8B). The NMDA-evoked calcium signals were blocked by MK-801 (NMDA receptor ion channel blocker) and ifenprodil (NR2B subunit specific antagonist) [29] in a dose-dependent manner, indicating the functional expression of NMDA receptors containing the NR2B subunit (Figure 8C and 8D).

These data suggest that the ESC-derived neuroectoderm retains the potential to differentiate into cortical neurons, expressing functional NR2B subunit-containing NMDA receptors.

## Discussion

Several growth factors have been described as potent self-renewal inducers for primate ESCs [4], [30]-[33]. The combination of FGF and activin signaling is the most common and effective culture condition for maintaining primate ESCs [4], [30], [32], [33]. Based on these observations, we formulated the MT-fCFA medium, a chemically defined medium supplemented with FGF and activin. Our culture system did not require small molecule inhibitors of growth factor signaling to maintain cells. Based on the balance between FGF and activin signaling, the culture system allows for maintaining cells in a more natural state. Depending on the cell strains used, the concentrations of FGF and activin in the culture medium can be adjusted to suit the particular requirements. Although complete dissociation of primate PSC clumps into single cells generally causes extensive cell death, the use of ROCK inhibitors such as Y-27632 has significantly improved single-cell plating efficiency [34]-[37]. We used thiazovivin as an inhibitor of ROCK activity in this culture system for better maintenance of the undifferentiated state than Y-27632 [19], [20].

We have shown that both CMK6_SFF_ and CMK970 cells cultured in the MT-fCFA medium have been maintained at an undifferentiated state and retained a normal karyotype (Figure 3) and pluripotency (Figure 2, 4 and S1). The cells grown under the MT-fCFA culture exhibited a higher proliferation rate than those grown under the conventional culture condition (Table 1). In particular, the cells have been grown as a uniform monolayer, and not as a colony (Figures 1A and 1B), although the reasons for this property are yet to be fully determined. On the other hand, in conventional or most feeder-free culture systems, primate PSCs usually expand as colonies, which are heterogeneous cell populations [10], [38], [39]. The uncertainty of desired differentiation and the restriction of large-scale propagation are mainly attributed to the difficulty in controlling the quality and growth of colonies. For example, Tsutsui et al. reported single-cell culture methods for screening the effects of small molecule combinations on PSC growth, but the cells were grown and assayed as colonies [8]. Chen et al. and Kunova et al. recently reported single-cell based monolayer culture methods, but their methods required MEF-CM and Matrigel [9], [10]. Matrigel is not an optimal substrate because it contains many unknown components [14], [15]. Furthermore, its cost is very high. MEF-CM also includes several unknown contaminants, which give rise to unstable experimental conditions and variable results. Preparing MEF-CM is very labor-intensive and time-consuming. In contrast, our culture system did not require Matrigel and MEF-CM. Furthermore, all of the culture methods mentioned above required preparing surface coatings in culture dishes or plates before cell plating [8]–[10]. We used fibronectin for promoting cell attachment to the culture dish bottom [4]. Unlike Matrigel and general extracellular matrix, pre-coating before cell plating was not necessary, which is labor-intensive and time-consuming (Figure S3). Thus, our culture system is not only a high-quality single-cell and feeder-free culture system, but is also a cost-effective, user-friendly culture system.

In our culture system, directed differentiation could be selectively initiated only by modifying growth factor signaling, i.e., removing FGF and/or activin from the MT-fCFA medium and using small molecule inhibitors, given the simple composition of the medium that did not include any unknown factors (Table 2, Figures 7 and S2). In addition, as an example, we have shown that ESC-derived neuroectodermal cells retain the potential to differentiate into cortical neurons (Figure 8). With respect to genetic manipulation, primate PSCs under conventional culture conditions had low transfection efficiencies and difficulty in clone isolation because of colony formation [34], [40]. We demonstrated that the stably transfected clonal cells were readily isolated without optimizing transfection conditions (Figure 5). The result indicates that the single-cell culture system allows for successful clonal isolation after gene transfer. Moreover, we confirmed that the culture system permits effective cryopreservation of single dissociated cells using the commercial freezing medium (Figure 6). It does not require a specific freezing apparatus and is easy to handle. The cryopreservation method enables freezing of large quantities of cells for both research purposes and clinical long-term storage.

In conclusion, no independent culture systems based on the MT-fCFA medium have been previously developed and characterized. The culture system offers not only an effective alternative for the robust maintenance of primate PSCs but also a general-purpose platform for studying the mechanisms of self-renewal and differentiation, facilitating both research and clinical applications of primate PSCs.

## Supporting Information

Figure S1 **Pluripotency of CMK970 cells grown under the MT-fCFA culture condition.** A. Immunocytochemical analyses show that CMK970 cells (P28) have a characteristic expression pattern of typical pluripotency factors, Nanog, Oct4, and Sox2 as well as that of cell surface markers, SSEA-4, TRA-1-60, and TRA-1-81, indicating their undifferentiated and pluripotent state. *Scale bar* = 100 µm. B. Flow cytometric analysis of Nanog, Oct4, and Sox2 co-expressing CMK970 cells (P33) under the MT-fCFA culture condition. Cells were co-stained with Alexa Fluor 647-conjugated anti-Nanog, Alexa Fluor 488-conjugated anti-Oct4, and PE-conjugated anti-Sox2 or the corresponding isotype controls.
(TIFF)

Figure S2 **Directional differentiation to mesendodermal cell lineages.** CMK6_SFF_ cells were treated in the MT-CDM medium in the presence of 10 ng/ml activin and 1 µM DM for 4 days. A combination of activin and DM in the MT-CDM medium generated cells that highly expressed the mesendoderm markers T/Brachyury. DM, dorsomorphin (BMP inhibitor). *Scale bar* = 100 µm.
(TIFF)

Figure S3 **Growth curves of CMK6_SFF_ cells in the MT-fCFA medium in the presence or absence of fibronectin and thiazovivin.** The single dissociated cells were resuspended in the MT-fCFA medium in the presence or absence of fibronectin and thiazovivin before plating. The growth curves were built from confluence measurements acquired at 2-h intervals. Values from each time point were averaged across 16 separate regions. Error bars represent 1 SD about the mean for 16 independent regions. FN, fibronectin. TZV, thiazovivin.
(TIFF)
