# Supplementary figures and images for: A Single-Cell and Feeder-Free Culture System for Monkey Embryonic Stem Cells
Source: PLoS One. 2014 Feb 5;9(2):e88346. doi: 10.1371/journal.pone.0088346 (PMC3915054; doi:10.1371/journal.pone.0088346)

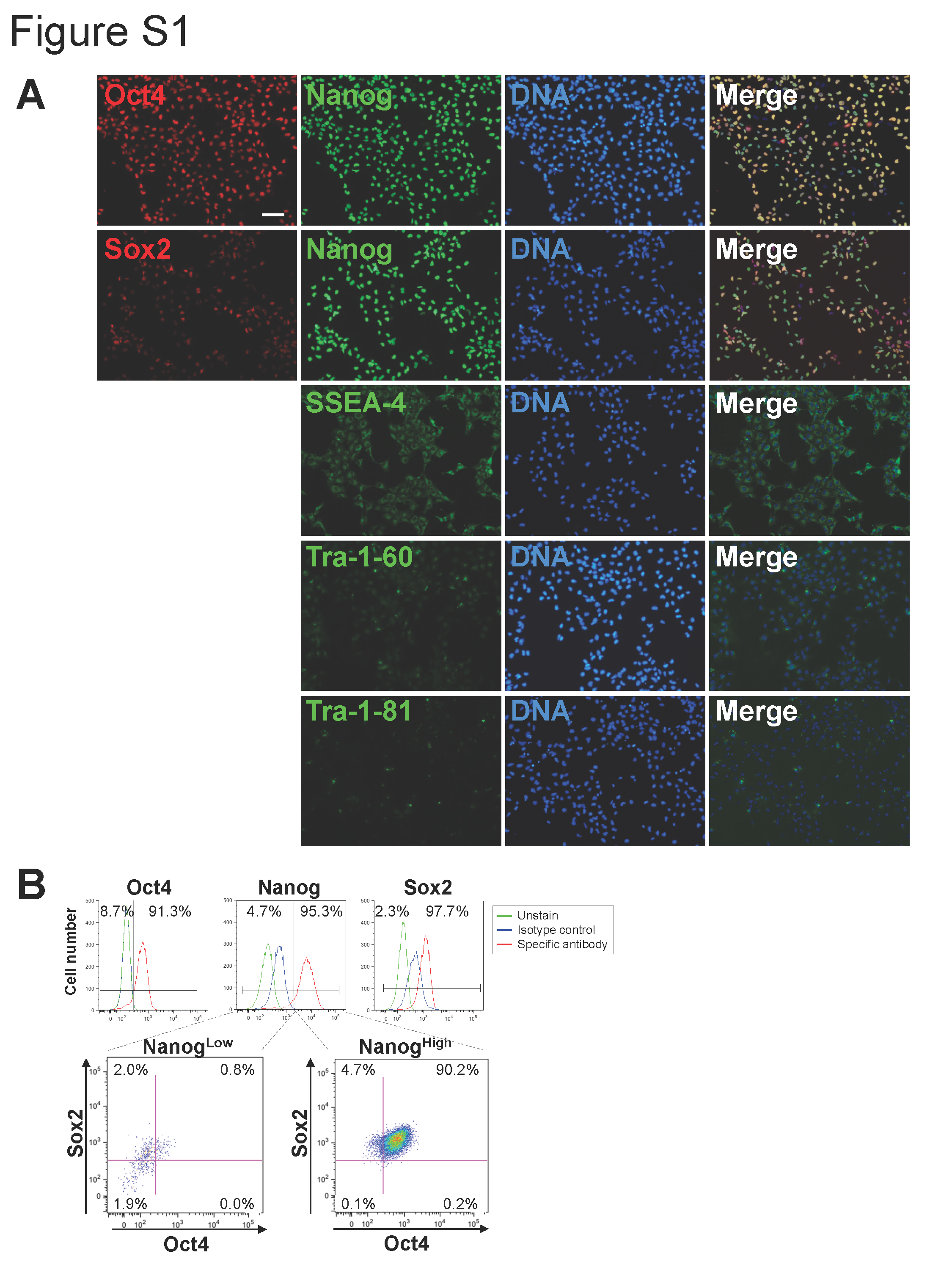

Supplement: Figure S1 — Pluripotency of CMK970 cells grown under the MT-fCFA culture condition. A. Immunocytochemical analyses show that CMK970 cells (P28) have a characteristic expression pattern of typical pluripotency factors, Nanog, Oct4, and Sox2 as well as that of cell surface markers, SSEA-4, TRA-1-60, and TRA-1-81, indicating their undifferentiated and pluripotent state. Scale bar = 100 µm. B. Flow cytometric analysis of Nanog, Oct4, and Sox2 co-expressing CMK970 cells (P33) under the MT-fCFA culture condition. Cells were co-stained with Alexa Fluor 647-conjugated anti-Nanog, Alexa Fluor 488-conjugated anti-Oct4, and PE-conjugated anti-Sox2 or the corresponding isotype controls. (TIFF) [file pone.0088346.s001.tiff]

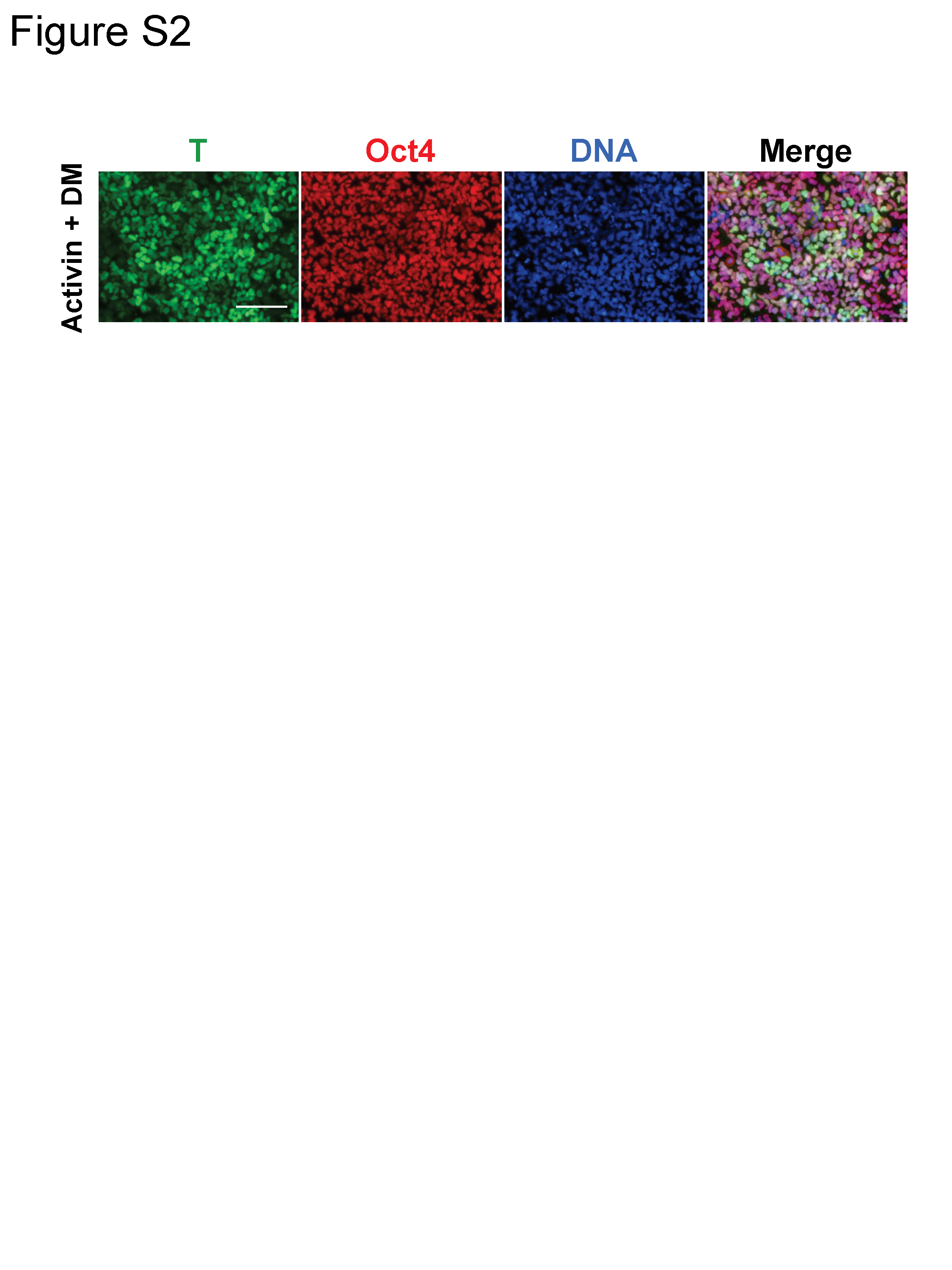

Supplement: Figure S2 — Directional differentiation to mesendodermal cell lineages. CMK6SFF cells were treated in the MT-CDM medium in the presence of 10 ng/ml activin and 1 µM DM for 4 days. A combination of activin and DM in the MT-CDM medium generated cells that highly expressed the mesendoderm markers T/Brachyury. DM, dorsomorphin (BMP inhibitor). Scale bar = 100 µm. (TIFF) [file pone.0088346.s002.tiff]

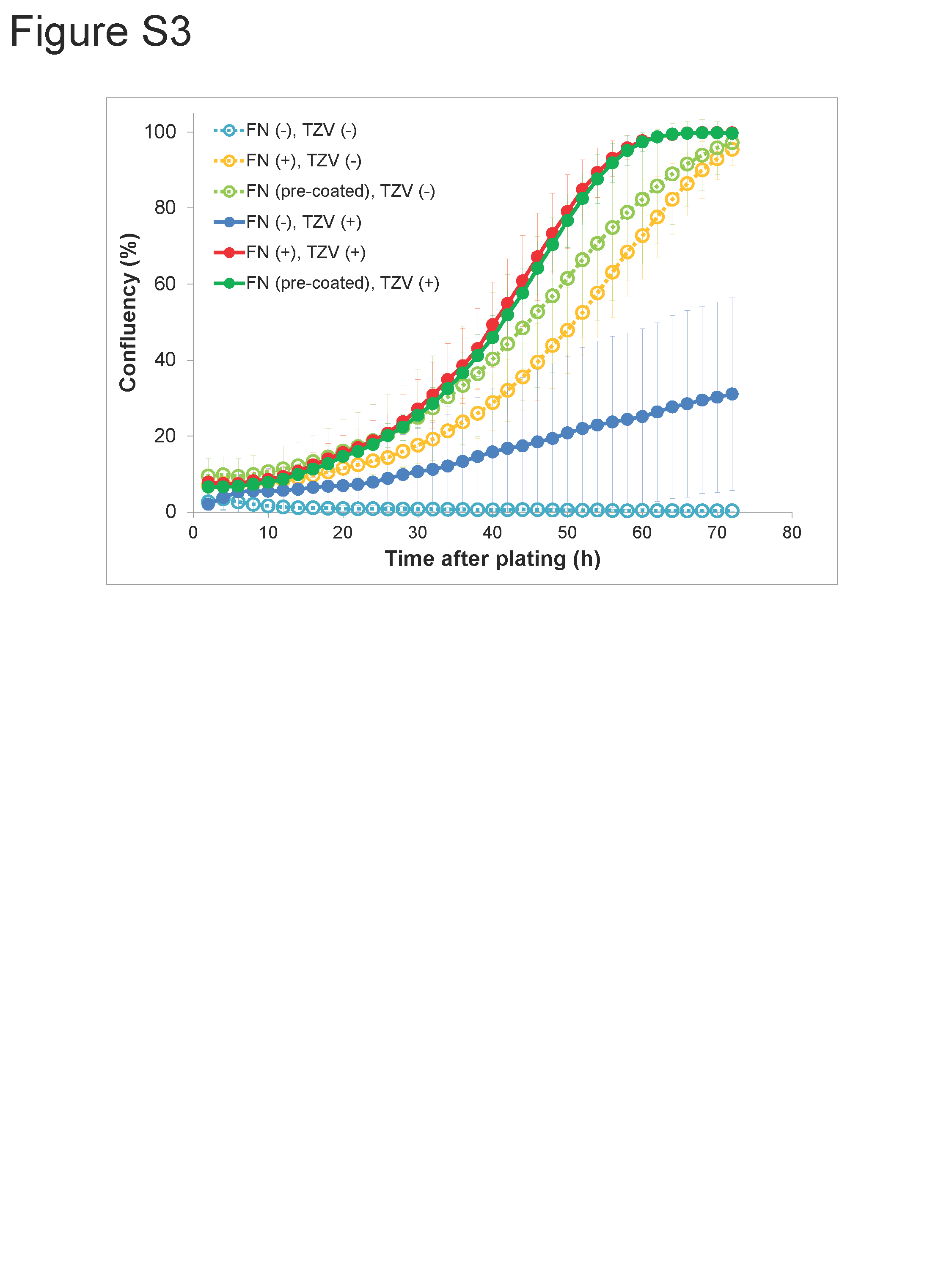

Supplement: Figure S3 — Growth curves of CMK6SFF cells in the MT-fCFA medium in the presence or absence of fibronectin and thiazovivin. The single dissociated cells were resuspended in the MT-fCFA medium in the presence or absence of fibronectin and thiazovivin before plating. The growth curves were built from confluence measurements acquired at 2-h intervals. Values from each time point were averaged across 16 separate regions. Error bars represent 1 SD about the mean for 16 independent regions. FN, fibronectin. TZV, thiazovivin. (TIFF) [file pone.0088346.s003.tiff]
